# Downregulation of homeobox gene Barx2 increases gastric cancer proliferation and metastasis and predicts poor patient outcomes

**DOI:** 10.18632/oncotarget.11260

**Published:** 2016-08-12

**Authors:** Yushuai Mi, Senlin Zhao, Chongzhi Zhou, Junyong Weng, Jikun Li, Zhanshan Wang, Huimin Sun, Huamei Tang, Xin Zhang, Xiaofeng Sun, Zhihai Peng, Yugang Wen

**Affiliations:** ^1^ Department of General Surgery, Shanghai General Hospital, School of Medicine, Shanghai Jiaotong University, 200080 Shanghai, China; ^2^ Department of Oncology and Department of Clinical and Experimental Medicine, Linkoping University, SE-581 85 Linkoping, Sweden; ^3^ Department of Pathology, Shanghai General Hospital, School of Medicine, Shanghai Jiaotong University, 200080 Shanghai, China; ^4^ Department of Pathology, Zhejiang Provincial People's Hospital, 310014 Hangzhou Zhejiang, China

**Keywords:** gastric cancer, Barx2, progression, prognosis, Wnt/β-catenin

## Abstract

Barx2 is a Bar family homeodomain transcription factor shown to play a critical role in cell adhesion and cytoskeleton remodeling, key processes in carcinogenesis and metastasis. Using quantitative real-time PCR, Western blotting, and immunohistochemistry, we found that Barx2 is expressed at lower levels in human gastric cancer (GC) tissues than in adjacent normal mucosa. In a multivariate analysis, Barx2 expression emerged as an independent prognostic factor for disease-free and overall survival. Kaplan-Meier survival analysis showed a trend toward even shorter overall survival in the patient group with Barx2-negative tumors, independent of advanced UICC stage and tumor relapse. Using *in vitro* and *in vivo* assays, we demonstrated that under normal conditions Barx2 inhibited GC cell proliferation and invasiveness through inhibition of the Wnt/β-catenin signaling pathway. These findings indicate that reduction or loss of Barx2 dis-inhibits GC cell proliferation and invasion, and that reduction in Barx2 could serve as an independent prognostic biomarker for poor outcome in GC patients.

## INTRODUCTION

Despite a dramatic decline in gastric cancer (GC) morbidity and mortality in recent years, GC remains the fifth most common cancer and third leading cause of cancer death worldwide [1–4]. Currently, the majority of newly diagnosed patients with GC present with locally advanced or metastatic disease [5]. Identification of specific genetic alterations and biomarkers associated with the cancer may facilitate earlier diagnosis and advances in GC treatment and allow implementation of individualized therapeutic regimens [6].

Altered expression of transcription factors is a common mechanism in carcinogenesis because of their wide reaching effects on cell processes such as proliferation, cell-cell adhesion, and motility. The Bar family of homeodomain factors is divided into two groups: the BarH-like group of BarHl1 and BarHl2/MBH and the Barx group, which includes Barx1 and Barx2 [7–9]. Located on human chromosome 11q25, the human Barx2 gene has four exons, ranging in size from 85 to 1099 bp and encodes a 254 amino acid homeodomain-containing protein, which binds optimally to the DNA consensus sequence YYTAATGRTTTTY [10, 11]. Barx2 is expressed in various epithelial tissues undergoing remodeling and regulates the expression of specific cell adhesion molecules: L1, Ng-CAM, N-CAM, and cadherin 6 [7, 8, 12, 13]. Moreover, Barx2 is required for adhesion and aggregation of mesenchymal cells [14].

In ovarian cancer, Barx2 is expressed in the ovarian surface epithelium, where it induces the expression of cadherin 6, a functional suppressor of ovarian cancer progression [15]. In primary hepatocellular carcinoma, low expression of Barx2 is significantly correlated with tumor size, tumor differentiation, clinical stage, metastasis, and relapse, serving as an independent biomarker for adverse survival outcomes. Furthermore, Zhang *et al.* demonstrated a significant negative relationship between the expression levels of Barx2 and markers of the epithelial-mesenchymal transition (EMT) [16]. On the other hand, in breast cancer, Barx2 increases the expression of both estrogen receptor–α gene (ESR1) isoforms, and modulates the expression of the estrogen-responsive genes SOX5, RBM15, Dynein, mortalin, and active matrix metalloproteinase-9 (MMP9) and the tissue inhibitor of metalloproteinase (TIMP) genes. Elevated expression of Barx2 inhibits cell growth, survival, and invasion pathways that are critical to breast cancer progression [17]. Barx2 expression has been observed in cells throughout the gut and in epithelial cells in the proliferative and differentiated regions of the stomach [18].

In this study, we examined Barx2 expression in a tissue microarray (TMA) of samples from 264 patients to evaluate the association between its expression level and clinicopathologic features in GC. *In vitro* and *in vivo* cell functional assays were used to explore the mechanism of Barx2 in carcinogenesis of GC and to reveal any clinicopathological significance or prognostic value of Barx2 in GC.

## RESULTS

### Expression pattern of Barx2 in GC tissues

**Figure 1:**
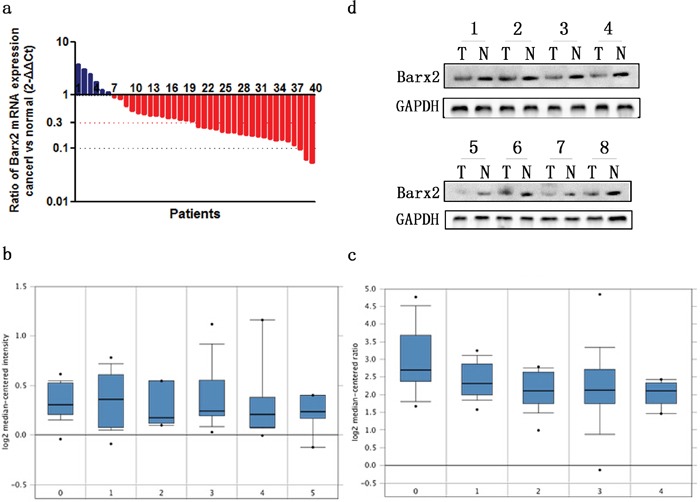
The expression of Barx2 in GC tissues and paired normal mucosa **a.** Quantitative real-time PCR detection of relative Barx2 expression in 40 human GC tissue specimens (T) and paired normal mucosa (N). A logarithmic 2^-ΔΔCT^ scale was used to represent the fold change in Barx2 mRNA expression in two independent microarray datasets from Oncomine database: Cho Gastric **b.** and Chen Gastric **c.**, grouped by no value (0), diffuse gastric adenocarcinoma (1), gastric adenocarcinoma (2), gastric intestinal type adenocarcinoma (3), gastric mixed adenocarcinoma (4) and gastrointestinal stromal tumor (5). **d.** Western blot analysis was used to detect Barx2 protein expression in 8 representative paired GC tissue samples, with GAPDH used as the loading control.

Forty paired specimens were randomly selected to explore the Barx2 expression level in GC by quantitative real-time PCR; 34 (85.0%) GC tissues showed decreased Barx2 mRNA expression compared to the matched normal mucosa (Figure 1a), consistent with two independent microarray datasets from the Oncomine database [19, 20] (Figure 1b–1c). Western blot (WB) analysis confirmed that Barx2 protein was down-regulated in the GC tissues compared with the corresponding normal mucosa (Figure 1d).

### Correlation between Barx2 expression and clinicopathological characteristics in GC

**Figure 2:**
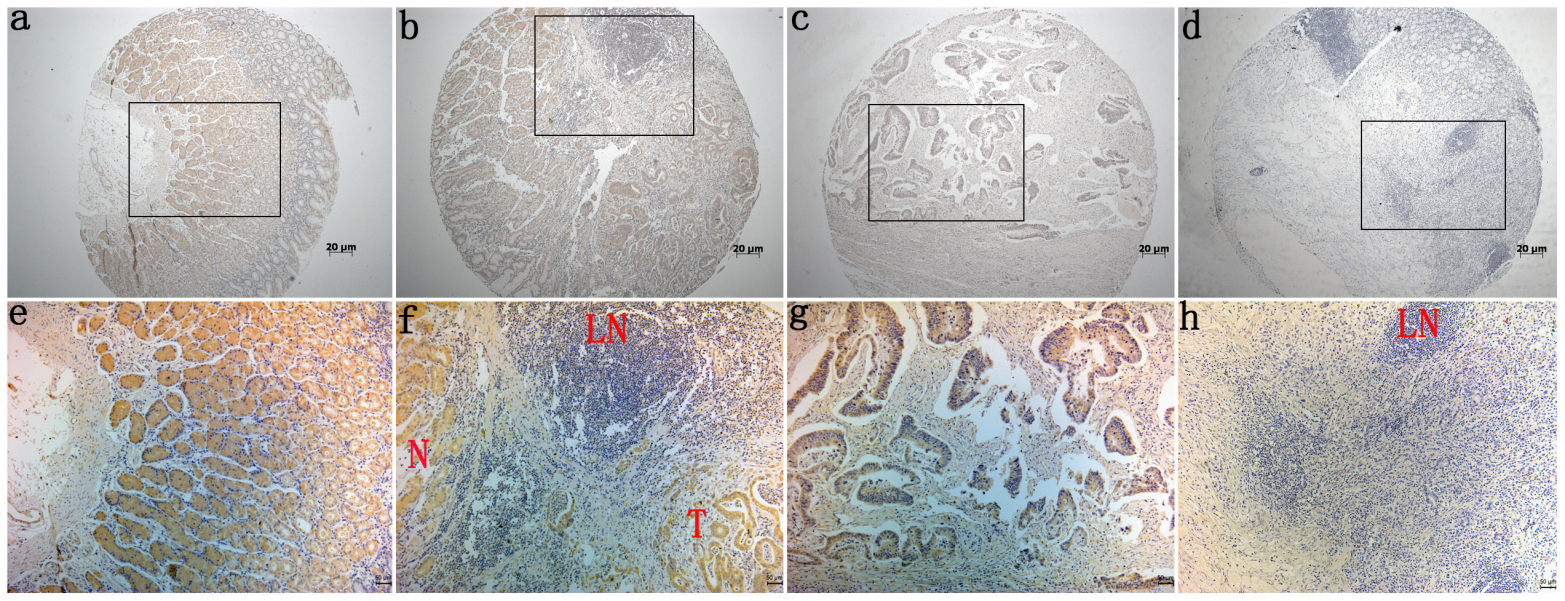
Immunohistochemical staining for Barx2 expression in normal gastric mucosa and cancerous tissues Barx2 protein expression was significantly lower in gastric cancer tissues compared with adjacent normal mucosa, with Barx2 staining observed both in the cytoplasm and nuclei of gastric cancer cells. **a, e:** Strong Barx2 staining in normal gastric epithelium; **b, f:** Intense Barx2 staining in well-differentiated gastric cancer; **c, g:** Weak Barx2 staining in moderately differentiated gastric cancer; **d, h:** Negative Barx2 staining in poorly differentiated gastric cancer; **N:** Strong Barx2 staining in normal gastric epithelium; **T:** Intense Barx2 staining in well-differentiated gastric cancer; **LN:** Negative Barx2 staining in lymph node; **a–d.** Original magnification: 50×; **e–h.** Original magnification: 200×.

Immunohistochemical (IHC) staining of Barx2 protein in a TMA which contained 264 cases of primary gastric cancer paired with normal mucosa and 104 lymph node metastasis (LNM) was used to investigate the relationship between Barx2 expression and the clinical characteristics of GC, summarized in Table 1. We found that Barx2 was expressed in normal gastric mucosa, and divided the patients into strong positive (205/264), weak positive (38/264), and negative staining (21/264) groups (Figure 2a, 2e). Barx2 was greatly reduced in the majority of GC tumor tissues with strong staining in only 17/264 (6.4%) specimens (Figure 2b, 2f), weak staining in 82/264 (31.1%) specimens (Figure 2c, 2g), and negative staining in 165/264 (62.5%) specimens (Figure 2d, 2h). These results further confirmed that the Barx2 expression level was down-regulated in GC tissues relative to adjacent normal mucosa (*P*=0.002). Furthermore, in the 88/104 (84.6%) available LNM specimens showed negative staining (Figure 2f, 2h-LN) and only 4 (3.8%) were strongly Barx2-positive.

**Table 1:** Expression of Barx2 in normal gastric mucosa, primary cancerous tissues and lymph node metastasis

Tissue sample	N	Expression of Barx2			*P*
		Negative (%)	Weak positive (%)	Strong positive (%)	
Normal mucosa	264	21 (8.0)	38 (14.4)	205 (77.7)	0.002^a^
GC tissue	264	165 (62.5)	82 (31.1)	17 (6.4)	0.002^b^
LNM tissue	104	88 (84.6)	12 (11.5)	4 (3.8)	0.020^c^

GC: gastric cancer; LNM: lymph node metastasis

a Significant difference in the expression of Barx2 between normal gastric mucosa and cancerous tissues

b Significant difference between GC tissues and LNM

c Significant difference between LNM and normal gastric mucosa

Table 2 summarizes the correlation between Barx2 expression level and GC clinicopathologic features. Decreased Barx2 expression was highly correlated with tumor invasion (pT stage, *P*<0.001), LNM (pN stage, *P*=0.012), distant metastasis (M stage, *P*=0.044), advanced UICC stage (*P*<0.001), vascular invasion (*P*=0.002), nerve invasion (*P*=0.03), and histological differentiation (*P*<0.001). On the other hand, no significant associations were found between Barx2 expression and age, gender, tumor location, or tumor size (*P*>0.05 for all, Table 2).

**Table 2:** Association between Barx2 expression and clinicopathological features in gastric cancer (n=264)

	N	Barx2 expression			*P*
		Negative (165)	Weak positive (82)	Strong positive (17)	
Age (yr)					0.476
<65	121	76(62.8%)	35 (28.9%)	10 (8.3%)	
>=65	143	89 (62.2%)	47 (32.9%)	7 (4.9%)	
Gender					0.175
Male	157	94 (59.9%)	55 (35.0%)	8 (5.1%)	
Female	107	71 (66.4%)	27 (25.2%)	9 (8.4%)	
Tumor location					0.147
Gastric fundus	11	5 (45.5%)	6 (54.5%)	0 (0.0%)	
Gastric corpus	123	74 (60.2%)	43 (35.0%)	6(4.9%)	
Pylorus	130	86 (66.2%)	33 (25.4%)	11 (8.5%)	
Tumor size (cm)					0.231
<3	77	42 (54.5%)	29 (37.7%)	6(7.8%)	
>=3	187	123 (65.8%)	53 (28.3%)	11 (5.9%)	
T stage					<0.001^*^
T 1	76	28 (36.8%)	37 (48.7%)	11 (14.5%)	
T 2	42	20 (47.6%)	20 (47.6%)	2 (4.8%)	
T 3	118	93 (78.8%)	21 (17.8%)	4 (3.4%)	
T 4	28	24 (85.7%)	4 (14.3%)	0(0.0%)	
N stage					0.012^*^
N 0	116	60 (51.7%)	43 (37.1%)	13 (11.2%)	
N 1	91	60 (65.9%)	28 (30.8%)	3 (3.3%)	
N 2	40	32 (80.0%)	7 (17.5%)	1 (2.5%)	
N 3	17	13 (76.5%)	4 (23.5%)	0 (0.0%)	
M stage					0.044^*^
M 0	254	155 (61.0%)	82 (32.3%)	17 (6.7%)	
M 1	10	10 (100.0%)	0 (0.0%)	0 (0.0%)	
UICC stage					<0.001^*^
I	95	38 (40.0%)	47 (49.5%)	10 (10.5%)	
II	48	24 (50.0%)	21 (43.8%)	3 (6.3%)	
III	89	75 (84.3%)	12 (13.5%)	2 (2.2%)	
IV	32	28(87.5%)	2 (6.3%)	2 (6.3%)	
Vessel invasion					0.002^*^
No	186	104 (55.9%)	69 (37.1%)	13 (7.0%)	
Yes	78	61 (78.2%)	13 (16.7%)	4 (5.1%)	
Nerve invasion					0.030^*^
No	206	122 (59.2%)	67 (32.5%)	17 (8.3%)	
Yes	58	43 (74.1%)	15 (25.9%)	0 (0.0%)	
Differentiation					<0.001^*^
High	47	10 (21.3%)	25 (53.2%)	12 (25.5%)	
Moderate	42	22 (52.4%)	19 (45.2%)	1 (2.4%)	
Low ^a^	175	133 (76.0%)	38 (21.7%)	4 (2.3%)	
Relapse					0.001^*^
No	145	78 (53.8%)	52 (35.9%)	15 (10.3%)	
Yes	119	87 (73.1%)	30 (25.2%)	2 (1.7%)	

a Low differentiation corresponds to signet ring cell carcinoma, mucinous adenocarcinoma, and poorly differentiated adenocarcinoma

* Significant difference

### Lower Barx2 expression predicts poorer clinical outcome in GC

**Figure 3:**
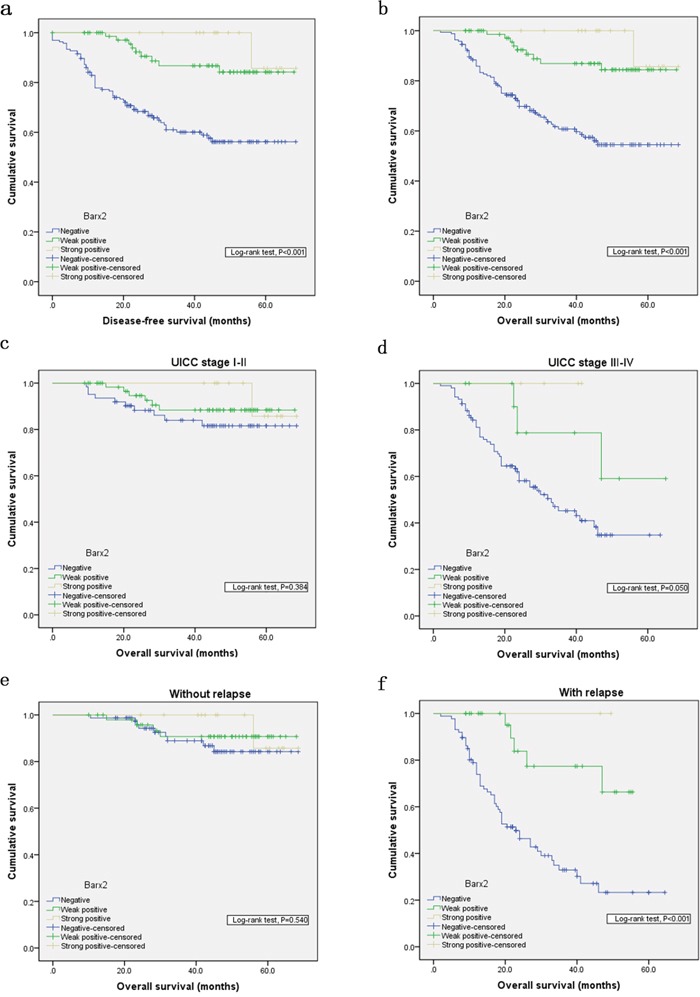
Kaplan-Meier analysis with a log-rank test of survival Disease-free survival **a.** and overall survival **b.** were significantly shorter in patients with Barx2-negative tumors than in those with Barx2-positive tumors (*** *P*< 0.001 for both, log-rank test). Comparisons of overall survival between patients with Barx2-negative tumors and those with Barx2-positive tumors in early UICC stage (I-II) cohort and in advanced UICC stage (III-IV) cohort **c-d.** and in patients with or without relapse **e-f.** P-values were calculated by log-rank test and *P*<0.05 was considered significant.

To explore the predictive role of Barx2 in GC patient survival, Kaplan Meier survival analysis with a log-rank test was used determine its relation to disease-free survival (DFS) and overall survival (OS). Patients with Barx2-negative tumors had a poorer DFS and OS rate than those with Barx2-positive tumors (*P*<0.001 for both, Figure 3a and 3b), suggesting that low Barx2 expression is a prognostic indicator for GC patients.

Univariate and multivariate survival analyses for DFS and OS were performed using the Cox proportional hazards model. In Univariate Cox proportional hazard analyses of DFS and OS, Barx2 expression, pM stage, UICC stage, vessel invasion, histological differentiation, and relapse (P<0.05 for all, Table 3) emerged as significant independent prognostic factors. Then, multivariate analysis was used to further analyze the factors found to be significant by univariate analysis. Barx2 expression, pM stage, vessel invasion, nerve invasion, histological differentiation, and relapse (P<0.05 for all, Table 3) were independent prognostic factors for DFS and OS. Collectively, these findings identify negative Barx2 expression as an independent prognostic biomarker for poor outcomes in patients with GC.

**Table 3:** Univariate and multivariate Cox proportional hazard models for overall survival and disease-free survival after surgery

	Overall survival				Disease-free survival			
	Univariate analysis		Multivariate analysis		Univariate analysis		Multivariate analysis	
	HR (95%CI)	*P*	HR (95%CI)	*P*	HR (95%CI)	*P*	HR (95%CI)	*P*
Age (yr)								
<65	-		-		-		-	
>=65	1.88 (1.10-3.2)	0.02	1.53 (0.94-2.45)	0.083	1.86 (1.09-3.17)	0.022	1.512 (0.940-2.432)	0.088
Gender								
Male	-				-			
Female	1.002 (0.603-1.666)	0.992			1.006 (0.605-1.671)	0.982		
Tumor location								
Gastric fundus	-				-			
Gastric corpus	2.02 (0.7-5.81)	0.192			2.01 (0.7-5.78)	0.195		
Pylorus	1.40 (0.83-2.36)	0.203			1.42 (0.84-2.38)	0.189		
Tumor size (cm)								
<3	-				-			
>=3	1.656 (0.91-3.01)	0.097			1.657 (0.91-3.01)	0.097		
T stage								
T 1	-		-		-		-	
T 2	2.78 (0.79-9.89)	0.113	1.56(0.66-3.69)	0.304	2.87 (0.81-10.18)	0.103	1.61 (0.66-3.85)	0.272
T 3	7.6 (2.71-21.3)	<0.001	2.59 (1.38-5.52)	0.005	7.54 (2.69-21.15)	<0.001	2.61 (1.33-5.01)	0.005
T 4	12.06 (3.93-37.04)	<0.001	3.74 (1.61-8.49)	0.002	12.2 (3.97-37.47)	<0.001	3.41 (1.49-8.45)	0.002
N stage								
N 0	-		-		-		-	
N 1	4.02 (1.8-9.2)	0.001	1.78 (0.97-3.58)	0.060	4.05 (1.81-9.05)	0.001	1.77 (0.96-3.23)	0.063
N 2	9.65 (4.24-21.96)	<0.001	3.86 (2.36-7.91)	<0.001	9.49 (4.17-21.59)	<0.001	3.76 (2. 01-7.16)	<0.001
N 3	16.91 (6.67-42.9)	<0.001	8.39 (3.77-17.43)	<0.001	18.15 (7.15-46.09)	<0.001	8.32 (4.03-18.47)	<0.001
M stage								
M 0	-		-		-		-	
M 1	3.08 (1.23-7.71)	0.016	4.56 (1.79-11.34)	0.001	3.41 (1.56-8.52)	0.009	2.64 (0.96-7.82)	0.008
UICC stage								
I	-		-		-		-	
II	6.80 (1.87-24.7)	0.004	0.35 (0.11-1.27)	0.101	6.87 (1.89-24.96)	0.003	3.56 (1.02-12.40)	0.007
III	13.46 (4.1-44.18)	<0.001	3.85 (1.69-6.04)	<0.001	13.36 (4.07-43.83)	<0.001	6.99 (3.23-30.68)	0.005
IV	32.07 (9.43-109.1)	<0.001	11.61 (5.62-21.73)	<0.001	34.58 (10.17-117.6)	<0.001	15.99 (6.48-60.92)	0.001
Vessel invasion								
No	-		-		-		-	
Yes	2.547 (1.542-4.206)	<0.001	2.18 (1.28-3.70)	0.004	2.548 (1.542-4.208)	<0.001	1.44 (0.79-2.66)	0.025
Nerve invasion								
No	-		-		-		-	
Yes	2.71 (1.61-4.56)	<0.001	2.16(1.29-3.60)	0.003	2.73 (1.63-4.6)	<0.001	1.37 (0.96-3.70)	0.052
Differentiation								
High	-		-		-		-	
Moderate	5.44 (1.16-25.63)	0.032	3.69 (0.86-16.29)	0.025	5.50 (1.17-25.91)	0.031	4.28 (0.72-22.12)	0.027
Low ^a^	8.51 (2.07-34.96)	0.003	5.56 (1.25-21.21)	0.014	8.58 (2.09-35.27)	0.003	7.80 (1.59-29.97)	0.016
Relapse								
No	-		-		-		-	
Yes	14.75 (6.35-34.29)	<0.001	10.23 (5.57-18.77)	<0.001	15.40 (6.63-38.81)	<0.001	10.98 (5.60-30.52)	<0.001
Barx2								
Negative	24.83 (11.61-54.87)	<0.001	15.50 (3.26-29.27)	0.001	24.18 (10.96-53.11)	<0.001	14.78 (3.11-27.96)	0.002
Weak	5.48 (3.25-9.24)	0.004	3.82 (2.04-9.27)	0.007	5.33 (3.19-8.96)	0.005	3.12 (1.97-8.95)	0.009
Strong	-							

HR hazard ratio; CI confidence interval

* *P*<0.05 indicate that the 95% CI of HR was not included

In order to confirm the correlation between decreased Barx2 expression and tumor metastases or local relapse independent of clinical stage, we performed further overall survival analysis according to UICC stages and tumor relapse. Interestingly, in patients with stage III-IV disease, decreased Barx2 expression was significantly associated with poorer OS (*P*=0.050, Figure 3d), while in patients with stage I-II, decreased Barx2 expression did not significantly affect OS (*P*=0.384, Figure 3c). There was similar trend toward shorter OS in patients with Barx2-negative tumors than those with Barx2-positive tumors with or without relapse (*P*<0.001 *vs. P*=0.540, Figure 3e-3f). In summary, decreased expression of Barx2 could serve as a novel independent prognostic biomarker for shorter overall survival independent of advanced clinical stage and tumor relapse.

### Barx2 inhibits GC cell proliferation, migration and invasion *in vitro*

**Figure 4:**
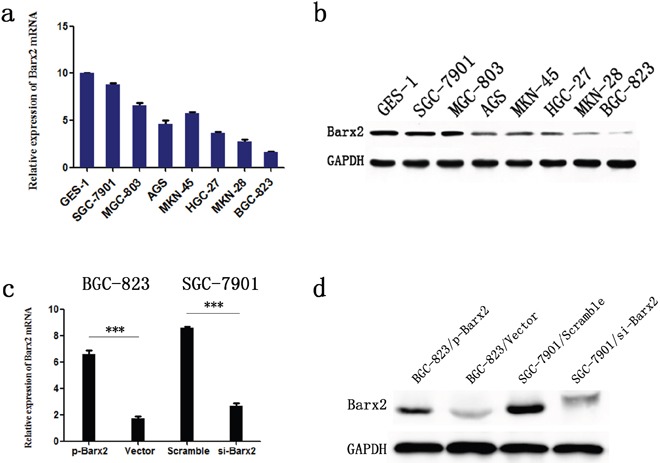
Barx2 expression in cell lines Barx2 mRNA levels **a.** and protein levels **b.** in gastric mucosa cell line (GES-1) and 7 GC cell lines. Barx2 mRNA and protein expression in BGC-823 and SGC-7901 cells transfected with Barx2 overexpression or downregulation vectors were validated using quantitative real-time PCR **c.** and Western blotting **d.** GAPDH was used to normalize mRNA and protein levels.

To investigate the effect of Barx2 reduction on cancer biological processes in GC, we first compared Barx2 expression in seven GC cell lines and one healthy gastric mucosa cell line. From these we selected the SGC-7901 cell line, which had the highest Barx2 expression, and the BGC-823 cell line, which showed the lowest Barx2 expression, for further studies (Figure 4a-4b). Then, we generated a high Barx2-expressing BGC-823 cell line and a Barx2 knock down SGC-7901 cell line and evaluated their Barx2 expression by qRT-PCR (Figure 4c) and WB (Figure 4d).

**Figure 5:**
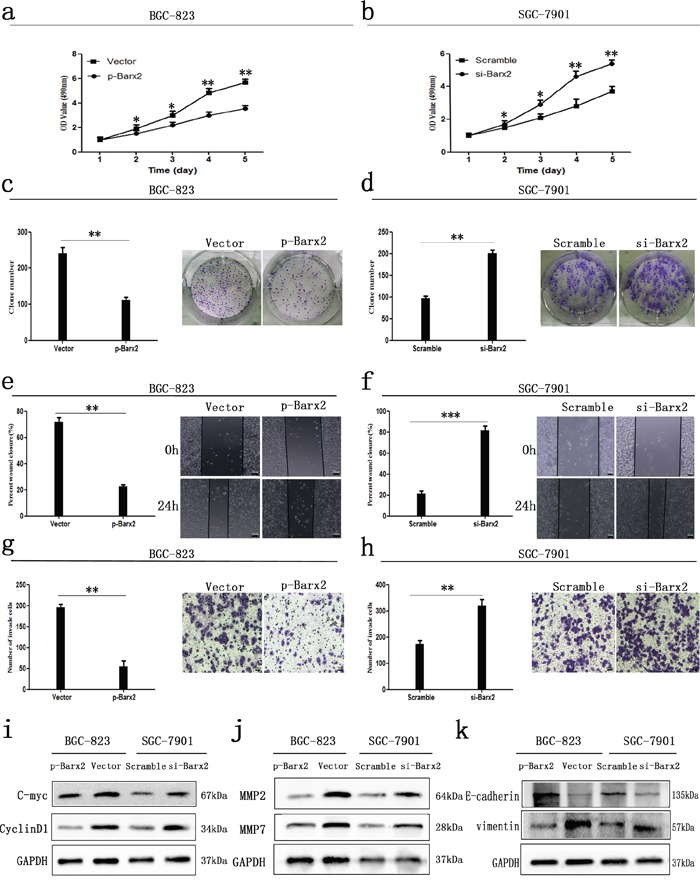
*In vitro* GC cancer cell functional assays Overexpression or knock down of Barx2 inhibited or elevated GC cells proliferation **a, b.**, colony formation **c, d.**, wound healing **e, f.**, migration and invasion ability **g, h.**, compared with their control group, respectively (* *P* <0.05; ** *P* <0.01; *** *P*<0.001). **e–f.** Original magnification: 100×; **g–h.** Original magnification: 200×. **i-k.** Western blot analysis of cell cycle-related proteins (c-myc and CyclinD1), biomarkers of cell invasion (MMP2 and MMP7), and EMT markers (E-cadherin and vimentin) in the Barx2 overexpressing or knockdown cells compared with their control groups.

CCK-8 assays showed that ectopic overexpression or knockdown of Barx2 significantly inhibited or increased GC cell proliferation, respectively, in a time-dependent manner, compared with the control group *in vitro* (*P*<0.05 for all, Figure 5a, 5b). Moreover, Barx2 overexpression or knockdown in GC cells reduced or elevated the GC cells’ colony formation ability compared with control cells, respectively (Figure 5c, 5d). Consistent with those observations, ectopic overexpression or knockdown of Barx2 resulted in decreased or increased expression of cell cycle-related protein c-myc and CyclinD1, respectively, which are indicators of cell proliferation (Figure 5i) [21]. Wound healing assays showed that overexpression or knockdown of Barx2 delayed or accelerated GC cell wound healing, respectively (*P*<0.05 for all, Figure 5e-5f). Transwell assays further demonstrated that overexpression or knockdown of Barx2 attenuated or strengthened GC cell migration and invasion, respectively (*P*<0.05 for all, Figure 5g-5h). These results were supported by levels of tumor invasion and metastasis-associated biomarkers (MMP2 and MMP7), which were decreased or increased in Barx2 overexpressing or downregulated GC cells, respectively (Figure 5j). Taken together, these data indicate that Barx2 inhibits GC cell proliferation, wound healing, migration, and invasion *in vitro*. Interestingly, we found that Barx2 expression level was associated with biomarkers of epithelial-mesenchymal transition (EMT), and down-regulated expression of Barx2 correlated with absence of E-cadherin and elevated levels of vimentin (Figure 5k), offering novel insight into the role of Barx2 in GC progression and prompting further study.

### Knockdown of Barx2 promotes tumorigenesis *in vivo*

**Figure 6:**
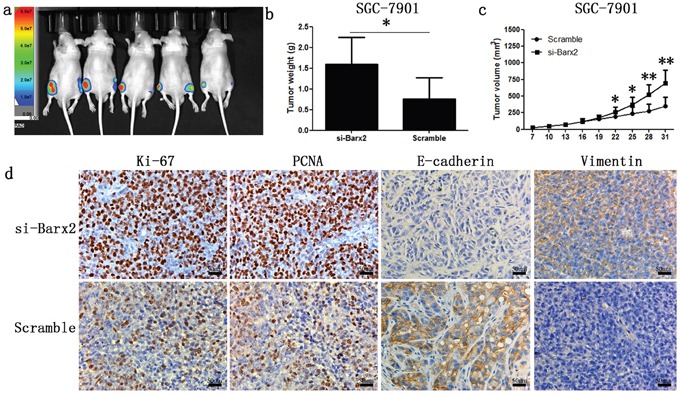
Knock-down of Barx2 promoted tumor formation ability of GC cells in nude mice (Left flank of the 5 nude mice: Barx2 knock-down; Right flank of the 5 nude mice: vector control) **a**. After four weeks, xenograft weight **b.** and volume **c.** curves were compared with controls (*n* =5, *P <0.05, **P <0.01). **d.** Immunochemical staining of xenograft tumors for biomarkers of cell proliferation (Ki-67 and PCNA) and EMT markers (E-cadherin and vimentin). Original magnification: 200×.

Cell functional assays previously demonstrated that Barx2 knockdown increases GC cells proliferation and colony formation ability *in vitro*. Further investigation *in vivo* showed that SGC-7901 cells with Barx2 knocked down generated larger subcutaneous xenografts, as measured by tumor weights and volumes in nude mice compared with the control (*P*<0.05, Figure 6a-6c). IHC staining revealed that tumor xenografts with Barx2 knocked down showed higher expression of the cell proliferation markers Ki-67 and PCNA than controls (Figure 6d), consistent with results of *in vitro* assays.

### Downregulation of Barx2 promotes the proliferation and invasion abilities of GC cells by activating the Wnt/β-catenin signaling pathway

**Figure 7:**
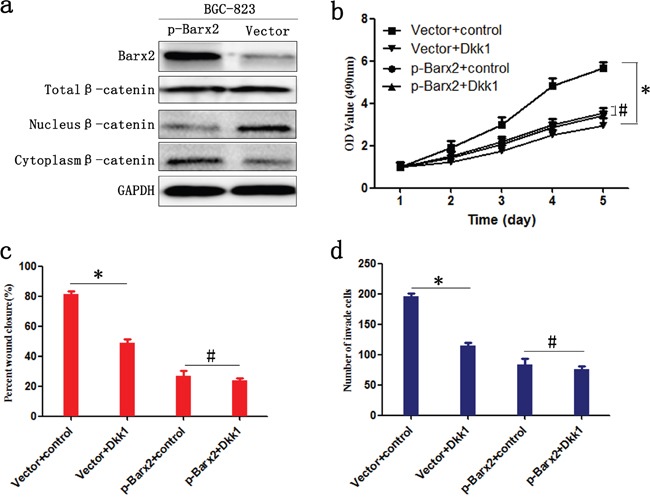
Barx2 inhibits the proliferation, migration, and invasion of GC cells by negatively regulating the Wnt/β-catenin signaling pathway **a.** WB analysis of the association between expression levels of Barx2 and β-catenin in total cellular, nuclear, and cytoplasmic fractions isolated from BGC-823 cells at 48 h after transfection with Barx2 overexpression or the control empty vector. BGC-823 cells with Barx2 overexpression or vector control were grown in media with or without 200 ng/ml inhibitor of Wnt signaling Dickkopf-related protein 1 (Dkk1); CCK-8 assays **b.**, wound healing **c.** and transwell assays **d.** were used to examine the GC cells’ proliferation, migration, and invasion ability, respectively. (* *P*<0.05, # *P*>0.05).

As downstream effectors of the Wnt/β-catenin pathway, c-myc, CyclinD1, MMP-2, and MMP-7 promote tumor cell proliferation, cell cycle, and migration [22, 23]. We have found a significant negative correlation between Barx2 and these Wnt signaling target genes (Figure 5i and 5j), which indicates that Barx2 may suppress GC cell proliferation, migration, and invasion by inhibiting the canonical Wnt/β-catenin pathway. To determine whether Barx2 regulates the Wnt/β-catenin signaling pathway in GC, we next examined Barx2 and β-catenin protein levels in GC cells by Western blot analysis, and found no association between Barx2 level and total cellular β-catenin. However, Barx2 overexpressing cells showed reduced nuclear β-catenin, an indicator of active Wnt/β-catenin pathway, and increased cytoplasmic β-catenin compared with control cells (Figure 7a), supporting a role for Barx2 as a negative regulator of the canonical Wnt/β-catenin pathway in GC cells.

We examined the downstream effect of increased Barx2 expression in GC using the Wnt signaling pathway inhibitor Dickkopf-related protein 1 (Dkk1). High Barx2-expressing BGC-823 cells with or without the addition of 200 ng/ml Dkk1. We found in Barx2 overexpressing cells proliferation, wound healing and invasion was not markedly inhibited by Dkk1, whereas cells transfected with empty vector were significantly inhibited (*P*< 0.05 for all, Figure 7b–7d). Taken together, these results suggest that Barx2 inhibition of GC progression is due, at least in part, to the transcriptional downregulation of Wnt/β-catenin target genes.

## DISCUSSION

In the present study, we demonstrate that Barx2 expression was much lower in GC tissues and paired LNM tissues than in corresponding human normal mucosa. This reduced tumor Barx2 predicted a poorer survival for GC patients after radical surgery. Moreover, *in vitro* assays verified that when present Barx2 inhibits GC cell proliferation, colony formation, wound healing, migration, and invasion. Further study *in vivo* demonstrated that Barx2 suppresses xenograft tumor formation and prohibits tumor tumor cell invasive activity in nude mice. In addition, we reveal that Barx2 suppresses Wnt/β-catenin signaling by decreasing expression of its downstream target genes, which mediate the pathway's tumor suppressor function in GC. All of these findings support the hypothesis that Barx2 acts as a tumor suppressor inhibiting GC cell proliferation and invasiveness.

Human Barx2 has 100% identity within the homeodomain to mouse Barx2. Sixty potential Barx2 target loci were identified, proximal to or within introns of genes involved in cytoskeletal organization, cell adhesion, growth factor signaling, transcriptional regulation, and RNA metabolism [24, 25]. Moreover, focal Barx2 expression was observed at sites undergoing epithelial-mesenchymal transition and its aberrant expression has been reported in some malignant cancers with poor prognosis, such as ovarian cancer [15], hepatocellular carcinoma [16], and breast cancer [17]. In our study, we demonstrate lower Barx2 mRNA and protein levels in GC tissues/cells than in corresponding normal mucosa/cells. Consistent with our qRT-PCR and WB results, positive Barx2 protein staining was more visible in normal gastric mucosa than in primary GC specimens (92.0% *vs.* 37.5%, *P*=0.002) and corresponding LNM tissue (92% *vs.* 15.4%, *P*=0.020) according to IHC staining in a TMA, suggesting that Barx2 takes part in the tumorigenesis and progression of GC.

Mutations and evolution involved in GC exert significant influence on clinical pathology [26, 27]. Of the various clinicopathologic characteristics assessed in the current study, TNM stage, UICC stage, distant metastasis, histological differentiation, and relapse were found to be strikingly associated with low Barx2 expression. Together with the trend of a poorer DFS and OS in Barx2-negative tumors than in Barx2-positive tumors, we conclude that the decreased Barx2 expression likely correlates with tumor invasion and metastasis and provides a novel biomarker for a highly malignant GC phenotype. Most importantly, for the first time, we conclude that negative Barx2 staining predicts poorer survival in the advanced UICC stage and tumor relapse patients.

Sellar *et al.* found that Barx2 expression correlates with the expression of cadherin 6 and inhibits ovarian cancer cells’ ability to invade Matrigel and to adhere to collagen IV-coated plates [17]. Barx2 may mediate ras/raf dependent transcription of the calcitonin gene via a ras/raf responsive promoter element and loss of *Barx2*-mediated differentiation may lead to loss of expression of the calcitonin marker, further resulting in MTC tumor progression [11, 28, 29]. In primary hepatocellular carcinoma, the expression of Barx2 had a negative correlation with markers of EMT, providing evidence that its low expression level in hepatocellular carcinoma was significantly correlated with tumor metastasis [16]. All of these reports suggest that Barx2 works as a tumor suppressor gene. However, as reported in breast cancer, Barx2 could also serve as an oncogene, promoting cell growth and invasion. Barx2 increased expression of both ESR1 isoforms, the estrogen-responsive genes (SOX5, RBM15 and Dynein), the tissue inhibitor of metalloproteinase (TIMP) genes (TIMP1 and TIMP3), and MMP-9, all of which promote breast cancer cell growth, survival, and invasion [17]. Taken together, Barx2 is indeed involved in tumor progression, but its diverse functions in different cancers may be paradoxical.

Homeodomain transcription factors drive development by regulating regional patterns of gene expression that control diverse cellular behaviors such as differentiation, proliferation, adhesion, migration, and apoptosis [30–32]. The varied expression levels of Barx2 in GC cell lines may be associated with the different invasion ability of the cell lines. Further verified by the higher expression of cell proliferation biomarkers Ki-67 and PCNA with IHC staining of the tumor xenografts, we demonstrated that Barx2 inhibits GC cell proliferation *in vitro* and *in vivo*. As high cell proliferation rates play important roles in cancer progression and cell cycle change affects cell proliferation [33–35], we also evaluated the levels of cell cycle-related proteins and found that overexpression or knockdown of Barx2 resulted in weakened or elevated expression of c-myc and CyclinD1, giving further assurance to the idea that Barx2 inhibits the viability of GC cells. Because matrix metalloproteinases (MMPs) have long been associated with cancer-cell invasion and metastasis [36], we verified that overexpression or knockdown of Barx2 led to decreased or increased expression levels of the biomarkers MMP-2 and MMP-7, consistent with results that Barx2 prohibits GC cell migration and invasion *in vitro*. Inactivation of E-cadherin has been demonstrated to be of causal importance in the adenoma-to-carcinoma transition in murine transgenic models [37] and in human familial gastric cancer E-cadherin is inactivated by mutation [38]. Interestingly, compared with the control cohort, the Barx2 knock-down cohort showed higher E-cadherin and lower vimentin expression in IHC staining of tumor xenografts, confirming our hypothesis that Barx2 acts as a cancer suppressor gene in GC.

The Wnt/β-catenin signaling pathway has emerged as a critical factor in stem cell biology, organogenesis, tissue homeostasis, and tumorigenesis [39–42]. β-Catenin, as a central downstream effector of Wnt signaling, plays a key role in the regulation of growth and development [43]. Because Barx2 has been reported to be involved in Wnt signaling [44], we explored the effect of Barx2 on the Wnt/β-catenin signaling pathway in GC carcinogenesis. We found a significant negative correlation between Barx2 and downstream target genes of the Wnt/β-catenin signaling pathway, such as c-myc, CyclinD1 and MMP-7. Barx2 inhibited nuclear β-catenin accumulation, an indicator of active Wnt/β-catenin pathway, in GC cells [45], further confirming that Barx2 inhibits Wnt/β-catenin signaling in GC. Inhibition of Wnt signaling using Dickkopf-related protein 1 (Dkk1) suppressed GC cell proliferation, wound healing, and invasion ability in cells transfected with empty vector significantly more than in cells with Barx2 overexpression, indicating that Wnt/β-catenin signaling is a key target of Barx2 during GC tumorigenesis.

In summary, this study provides the first insight into the clinical significance of the Barx2 in human GC. We demonstrate that Barx2 expression was down-regulated in GC samples and that Barx2 downregulation promotes proliferation, invasion, and metastasis using *in vitro* and *in vivo* assays. Finally, we reveal that Barx2 inhibits Wnt/β-catenin signaling pathway by decreasing expression of its downstream target genes in GC, highlighting its importance in altered cell signaling leading to GC carcinogenesis and progression.

## MATERIALS AND METHODS

### Patients and tissue specimens

A total of 264 GC patient samples were included at the time of diagnosis from patients treated at general surgery departments of the Shanghai Jiaotong University affiliated Shanghai General Hospital between 2004 and 2009. There were 157 males and 107 females with a mean age of 66 years (range 27-89 years). Written informed consent was obtained from all subjects and the research was carried out according to the World Medical Association Declaration of Helsinki. Overall survival (OS) and disease-free survival (DFS) rates were defined as the interval from the initial surgery to clinically or radiologically proven recurrence/metastasis and death, respectively.

All patient-derived specimens were collected and archived under protocols approved by the institutional review boards of Shanghai General Hospital Affiliated to Shanghai Jiao Tong University. Procedures were carried out in accordance with approved guidelines. Frozen in liquid nitrogen or formalin-fixed, paraffin-embedded cancer tissues and their paired adjacent normal mucosa were collected immediately after surgical resection, for subsequent RNA extraction or immunohistochemical staining. All diagnoses were confirmed by at least two certified pathologists, and the tumor grade and stage classification was based on pathological findings according to the International Union Against Cancer guidelines.

### Quantitative real-time PCR

Total RNA was isolated from primary tumor tissues, adjacent normal mucosa, and cell culture using TRIzol reagent (TaKaRa, Japan) according to the manufacturer's instructions. RevertAid™First Strand cDNA Synthesis Kit (Fermentas, USA) was used to reverse transcribe 2 ug of RNA according to the manufacturer's recommendations. Quantitative real-time PCR assays were performed with 4 μl of cDNA (1:10 dilution) and SYBR green (TaKaRa) in a total volume of 20 μl using the ABI 7900 Real-time PCR System (ABI, USA). The primers used for qRT-PCR were: Barx2, sense 5′-ATG ATC GAC GAG ATC CTC TC-3′ and antisense 5′-GCT TAA TGG TGG GGG TTC CG-3′; GAPDH, sense 5′-GGG AAG GTG AAG GTC GGA GT-3′ and antisense 5′-GGG GTC ATT GAT GGC AAC A-3′. Relative quantities (Δ cycle threshold (Ct) values) were obtained by normalizing to glyceraldehyde-3-phosphate dehydrogenase (GAPDH). Each PCR product was run in triplicate, and the relative Barx2 mRNA level was calculated by 2^-ΔΔCt^.

### Western blot analysis

Tissue and cell lysates were extracted using RIPA lysis buffer with the protease inhibitor phenylmethanesulfonyl fluoride (Beyotime Biotechnology, Jiangsu, China) Protein concentration was measured using the BCA protein assay kit (Beyotime Biotechnology) according to the manufacturer's instructions. Equivalent amounts of protein (30 ug) were separated on 10% SDS-PAGE gel and then transferred onto PVDF membranes (Millipore, Billeria, MA) using standard protocols. Membranes were blocked in 5% skim milk in TBST buffer for 1.5 h at room temperature, followed by incubation with primary antibodies at 4°C overnight. After incubation with a secondary antibody for 2 h at room temperature, proteins were detected using ECL regent (Millipore, Billeria, MA). Primary antibodies specific to Barx2 (1:200), GAPDH (1:1000), MMP7 (1:500) were purchased from Santa Cruz Biotechnology (Santa Cruz, CA, USA), and c-myc (1:000), CyclinD1 (1:1000), MMP2 (1:000), E-cadherin (1:800), vimentin (1:800), β-catenin (1:1000) were purchased from Cell Signaling Technology (Massachusetts, USA).

### TMA construction and immunohistochemical staining

TMA construction was undertaken as reported previously [46]. The expression of Barx2 in the TMA and tumor samples taken from nude mice were tested using standard immunohistochemical methods, and the staining intensity for Barx2 was scored as: 0 (negative), 1 (weak), 2 (moderate), and 3 (strong). Staining area was scored as: 0 (negative staining), 1 (less than 10% positive staining), 2 (10-50% positive staining), and 3 (50-100% positive staining). The sum of staining intensity and staining area scores provided the overall score, which was divided into three groups: 0–2, negative expression; 3–4, weak expression; and 5–6, strong expression [47, 48]. The corresponding primary antibodies were: Barx2 (1:100, Santa Cruz Biotechnology, Santa Cruz, CA, USA), Ki-67 (1:500, Cell Signaling Technology, Massachusetts, USA), PCNA (1:400, Cell Signaling Technology, Massachusetts, USA), E-cadherin (1:200, Cell Signaling Technology, Massachusetts, USA) and Vimentin (1:400, Cell Signaling Technology, Massachusetts, USA).

### Cell culture and transfection

Human GC cell lines AGS, HGC-27, BGC-823, MKN-45, MGC-803, MKN-28, SGC-7901, and the healthy human gastric mucosa cell line GES-1 were all obtained from the Type Culture Collection of the Chinese Academy of Sciences (Shanghai, China). All cell lines were cultured in 1640 medium supplemented with 10% FBS (Gibco, USA), under a humidified atmosphere containing 5% CO_2_ at 37°C.

Both small interfering RNA (siRNA) specially targeting Barx2 and pcDNA3.1-Barx2 plasmid for gene overexpression test and their control sequences were obtained from Biolink Biotechnology Co. (Shanghai, China). GC cells were transfected using Lipofectamine 2000 following the manufacturer's instructions.

### Cell proliferation and plate colony formation assays

Cell proliferation assay was evaluated with Cell Counting Kit-8 (CCK-8) assay according to the manufacturer's instructions. Cell viability was confirmed by measuring the absorbance at 490 nm on a Gen5 microplate reader (BioTek, Vermont, USA) at the appropriate time (1, 2, 3, 4, and 5 days).

For plate colony formation assays, transfected cells were seeded in six-well plates (800 cells/well) and cultured at 37°C under a humidified atmosphere containing 5% CO_2_ for 14 days. Following fixation by methyl alcohol for 15 min, cells were stained with 0.1% crystal violet solution for 20 min. Colonies were then counted and photographed. All assays were independently performed in triplicate.

### Wound healing and transwell assays

Transfected cells were trypsinized and seeded into 6-well plates (1.0 × 10^5^ cells/well). Upon reaching the exponential growth phase, cells were wounded by a sterile pipette tip and then washed with PBS. Images were captured at 0, 12, 24, and 48-h intervals, and wound widths were quantified and compared to baseline values.

The transwell 24-well Boyden chamber (Corning, USA) with 8.0 μm pore size polycarbonate membrane was used for the cell migration (without Matrigel) and invasion assays (with Matrigel) assays according to the manufacturer's protocol. Briefly, each group of cells (5 ×10^4^/chamber) was plated in the upper chambers in 200 ul serum-free media for 36 h, while the bottom chambers contained 600 ul media supplemented with 10% fetal bovine serum (FBS) as a chemoattractant. Cells that migrated and invaded to the reverse side of chamber inserts were fixed by methyl alcohol and stained with 0.1% crystal violet. Experiments were carried out in triplicate.

### Tumor formation assay in nude mice

The *in vivo* assay using nude mice was approved by the Institutional Animal Care and Use Committee of Shanghai Jiaotong University Affiliated Shanghai General Hospital. Four-week-old male BALB/C nude mice were purchased from Shanghai Research Center for Model Organisms and housed under pathogen-free conditions in the animal experiment center of Shanghai General Hospital. Stable cell lines SGC-7901/si-Barx2 and SGC-7901/Scramble were constructed by Biolink Biotechnology Co. (Shanghai, China). We subcutaneously inoculated 1 × 10^7^ cells in 200 μL RPMI-1640 medium into the left or right flanks of nude mice, respectively. Tumor volumes were measured weekly using an In-Vivo Imaging System (IVIS; Xenogen). After four weeks, the mice were euthanized. Xenografted tumor tissue samples were separated, weighted and embedded in paraffin. All animal protocols were approved by Shanghai Jiaotong University Affiliated Shanghai General Hospital Animal Care.

### Statistical analysis

Data analysis were carried out using the SPSS 22.0 statistical software package (SPSS, Chicago, IL, USA). Differences of Barx2 mRNA expression between GC tissues and adjacent normal mucosa were estimated by the Student's T-test. The χ^2^ test or Fisher's exact test was appropriately used to determine the statistical significance between Barx2 expression and clinicopathological variables. Survival curves were calculated by the Kaplan–Meier method with the log-rank test employed for the comparison of differences. The hazard ratio (HR) with 95% confidence interval in the Cox proportional hazards regressions were applied to estimate hazard risk of individual factors for DFS and OS. For all tests, *P*-value<0.05 was considered to be statistically significant.
